# An Automatic Modulation Recognition Method Based on the Multimodal Kernel Harmonic Feature Fusion Network

**DOI:** 10.3390/s25206352

**Published:** 2025-10-14

**Authors:** Qiancheng Zhang, Hongbing Ji, Lin Li

**Affiliations:** School of Electronic Engineering, Xidian University, Xi’an 710071, China; acts_qczh@163.com (Q.Z.); lilin@xidian.edu.cn (L.L.)

**Keywords:** automatic modulation recognition, impulsive noise, time–frequency analysis, multimodal feature fusion, kernel space mapping, deep learning

## Abstract

In increasingly complex electromagnetic environments, wireless communication systems face the severe challenge of non-Gaussian impulse noise. The moments of impulse noise tend toward infinity, reducing the distinguishability of signal features and thereby limiting improvements in signal modulation recognition rates. First, a time–frequency analysis method based on kernel space mapping is proposed to improve the distinguishability of time–frequency features in signals under impulse noise. On this basis, a multimodal kernel harmonic feature fusion network is constructed, combining convolutional neural networks and graph convolutional networks to extract and fuse kernel harmonic features from three modalities to achieve robust and accurate modulation recognition. The simulation results show a generalized signal-to-noise ratio of −2 dB, and the modulation recognition rate reaches 93.5%.

## 1. Introduction

With the rapid advancement of wireless communication technologies, automatic modulation recognition has played an increasingly significant role in fields such as spectrum monitoring, cognitive radio, and military communications [1,2]. However, widespread non-Gaussian impulse noise in complex electromagnetic environments, such as electromagnetic pulses and atmospheric discharge noise, poses severe challenges to this technology. This type of noise exhibits pronounced peaks and heavy-tailed distributions, with its second-order and higher-order statistics diverging. It disrupts the amplitude, phase, and power spectrum structure of signals, causing significant degradation in the performance of traditional modulation recognition methods based on Gaussian assumptions.

From the perspective of signal non-stationarity differences, existing modulation recognition methods primarily fall into two technical categories: cyclostationary feature analysis and time–frequency feature analysis. Cyclostationary feature methods analyze signals exhibiting periodic statistical variations through cyclic autocorrelation functions or cyclic spectra [3,4]. However, impulse noise disrupts the signal’s implicit periodic structure, reducing the discernibility of cyclostationary features. To address this, the authors in [5,6] propose the Cyclic Correlation Entropy (CCE) method, aiming to extract robust cyclic stationary features through high-dimensional space mapping. However, the second-order statistical components in correlation entropy computation correspond to the second harmonic in the cyclic frequency domain rather than the fundamental component with higher energy. This limits CCE’s ability to extract core modulation information, thereby affecting the upper limit of signal recognition performance. The GCMKF method [7] employs kernel space mapping techniques to extract cyclostationary features in high-dimensional spaces, demonstrating effective suppression of impulse noise. These signal recognition methods based on cyclostationary features exhibit limited analytical capability for non-periodic time-varying signals.

Time–frequency distribution methods identify non-stationary signals by capturing instantaneous frequency variation information. Recent studies extensively employ deep learning models to automatically extract time–frequency features. For instance, Refs. [8,9] utilize convolutional neural networks (CNNs) and Transformer frameworks, respectively, to identify modulated signals from time–frequency representations generated by short-time Fourier transforms (STFTs). The research in [10] utilizes convolutional networks to extract time–frequency distribution features, such as the Smooth Pseudo-wigner–Ville Distribution (SPWVD) and the Bern–Jordan Distribution (BJD) for signal classification. However, the spike characteristics of impulse noise generate sudden high-energy points in the time–frequency plane, while its moment divergence characteristics elevate the noise baseline, severely interfering with the extraction of effective signal time–frequency features. Modulation identification methods based on fractional low-order statistics (FLOSs) and time–frequency images [11] lack adaptive capability in practice due to their reliance on manually set thresholds or prior knowledge of noise. The research in [12] employs time–frequency image denoising techniques to suppress pulse interference. However, this method risks distorting the effective signal components while filtering noise, particularly destroying modulation features under low signal-to-noise ratios, which leads to reduced recognition accuracy.

To this end, an automatic modulation recognition method based on a multimodal kernel harmonic feature fusion network is proposed. This method enhances feature robustness through kernel space mapping technology and integrates time–frequency features, cyclostationary features, and kernel sequence features to recognize different signal types. The main contributions of this paper are as follows:

- A time–frequency analysis method based on kernel space mapping is proposed. By incorporating kernel space mapping technology into time–frequency analysis, the discernibility of time–frequency features in pulsed noise environments is improved, solving the performance degradation issue in traditional methods under such conditions.
- A multimodal kernel harmonic feature fusion network was constructed. This network fuses three types of modal information, including time–frequency features, cyclostationary features, and kernel space mapping sequences, solving the problem that a single modality struggles with, comprehensively characterizing complex modulation characteristics. It employs a graph for local–global joint modeling of signals, comprehensively enhancing the network’s feature representation capability.

The rest of this paper is organized as follows: Section 2 introduces the impulse noise model and the fundamentals of the Choi–Williams distribution (CWD); Section 3 presents the proposed time–frequency analysis method based on kernel space mapping and the multimodal kernel harmonic feature fusion network (MKHFFN); Section 4 analyzes the performance of the proposed method through simulations; and Section 5 summarizes the work.

## 2. Related Work

### 2.1. Noise Model

The symmetric alpha-stable (SαS) distribution is often used for impulse noise modeling [13], usually represented by its characteristic function.

(1) $$\varphi (\lambda ,\;\gamma ,\alpha )=\exp\left\{-\gamma {\left|\lambda \right|}^{\alpha }\right\}$$

where

(2) γ≥0,0<α<2

γ is the dispersion coefficient, similar to the standard deviation under the Gaussian distribution. α is the characteristic exponent. The larger its value is, the stronger the sub-Gaussian degree and impulsivity. Only if α=2 is the SαS transformed into a Gaussian distribution. When 0<α<2 (sub-Gaussian distribution), there is

(3) $$E(|x{|}^{p})\left\{\begin{array}{c} =\infty ,\mathrm{if}\;p\geq \alpha \\ <\infty ,\mathrm{if}\;0\leq p<\alpha \end{array}\right.$$

A Sub-Gaussian SαS distribution does not have finite second-order and higher-order statistics. What is worse is that their first-order moment is also infinite when 0<α≤1. This is the root cause of the severe performance degradation in existing methods. The SNR defined under the Gaussian distribution is also not suitable to describe the SαS distribution, and the Generalized SNR (GSNR) is redefined to describe the noise environment.

(4) $$GSNR=10\mathrm{lg}(P_{x}/\gamma )$$

where $P_{s}$ is the signal power.

### 2.2. Choi–Williams Distribution

The bilinear time–frequency distribution exhibits higher time–frequency energy concentration compared to the linear time–frequency distribution, thus finding extensive application in signal recognition. For a given signal, its general expression for Cohen’s Distribution (CD) can be expressed as

(5) $$CD_{x}(t,f)=\int_{-\infty }^{\infty }\int_{-\infty }^{\infty }AF_{x}(t,\nu )\phi (\tau ,\nu )e^{-j(\nu t+f\tau )}d\tau d\nu$$

where $AF_{x}(t,\nu )$ is the fuzzy function, τ is the delay, and ν is the Doppler shift. Let the kernel function be $\phi (\tau ,\nu )=\exp\left(-\alpha {\left(\tau \nu \right)}^{2}\right)$, and the expression for the Choi–Williams Distribution (CWD) is obtained by

(6) $$CWD(t,f)=\int_{-\infty }^{\infty }\int_{-\infty }^{\infty }\kappa_{tf}(t,\tau ,\mu )s(t+\tau /2)s(t-\tau /2)e^{-j2\pi f\tau }dud\tau$$

where $\kappa_{tf}\left(t,\tau ,\gamma \right)=\frac{1}{\sqrt{4\pi \alpha \tau^{2}}}\exp\left(-\frac{{\left(t-u\right)}^{2}}{4\alpha \tau^{2}}\right)$. According to Equation (3), under impulse noise conditions, the CWD exhibits significantly diminished feature representation capability due to its second-order statistical moment becoming infinitely large. Impulse noise not only generates sudden high-energy points in the time–frequency plane and elevates the noise baseline, but also readily couples with signal components, producing spurious cross-terms interference that severely compromises the quality of time–frequency representations. Therefore, this paper proposes a kernel space mapping-based CWD to enhance the discriminability of the time–frequency feature.

## 3. Proposed Method

### 3.1. The Kernel-Based Choi–Williams Distribution

The kernel function method transforms the original data into a higher-dimensional space through an implicit mapping function φ(⋅), thereby converting the linearly inseparable problem into a separable one. The method achieves this transformation solely by defining the kernel function κ(x,y)=<φ(x),φ(y)>, without explicitly constructing complex mapping functions. Inspired by this concept, this paper innovatively introduces the kernel method into the field of time–frequency analysis, proposing a kernel-based Choi–Williams distribution (KCWD). It can be expressed as

(7) $$KCWD(t,f)=\int_{-\infty }^{\infty }\int_{-\infty }^{\infty }\kappa_{tf}(t,\tau ,\gamma )\kappa_{s}\left(s(t+\tau /2),s(t-\tau /2)\right)e^{-j2\pi f\tau }dud\tau$$

where

(8) $$\kappa_{s}(x,y)={\langle \Phi \left(x\right),\Phi \left(y\right)\rangle }_{F}=\tan h(\omega x^{T}y)$$

The kernel function $\kappa_{s}(x,y)$ maps the correlation function to a higher dimension, where ω>0. It should be noted that although both $\kappa_{s}(x,y)$ and $\kappa_{tf}(t,\tau ,\gamma )$ are called kernel functions, their roles differ. The kernel $\kappa_{tf}(t,\tau ,\gamma )$ constrains the two-dimensional distribution in time and frequency, reducing cross-frequency components, functioning similarly to a window function. The kernel $\kappa_{s}(x,y)$, however, was introduced to suppress impulse noise in high-dimensional space and enhance signal feature distinguishability. $\kappa_{s}(x,y)$ can be expanded as

(9) $$\begin{array}{l} k_{s}(s(t+\tau /2),s(t-\tau /2))\;\\ =\frac{e^{\omega s(t+\tau /2)s(t-\tau /2)}-e^{-\omega s(t+\tau /2)s(t-\tau /2)}}{e^{\omega s(t+\tau /2)s(t-\tau /2)}+e^{-\omega s(t+\tau /2)s(t-\tau /2)}}\\ =\frac{2}{e^{\omega s(t+\tau /2)s(t-\tau /2)}+e^{-\omega s(t+\tau /2)s(t-\tau /2)}}\sum_{n=1}^{\infty }\frac{{\left(\omega s(t+\tau /2)s(t-\tau /2)\right)}^{2n-1}}{\left(2n-1\right)!}\\ =A(\omega ,t,\tau )\sum_{n=1}^{\infty }B(\omega ,n){\left(s(t+\tau /2)s(t-\tau /2)\right)}^{2n-1} \end{array}$$

where

(10) $$A(\omega ,t,\tau )=2/\left(e^{\omega s(t+\tau /2)s(t-\tau /2)}+e^{-\omega s(t+\tau /2)s(t-\tau /2)}\right)$$

and

(11) $$B(\omega ,n)=\omega^{2n-1}/(2n-1)!$$

A(ω,t,τ) exhibits Gaussian decay, enabling adaptive suppression of strong pulse interference based on the amplitude of the input signal. The kernel function $k_{s}(s(t+\tau /2),s(t-\tau /2))\;$ constructs a high-dimensional feature mapping space that fully preserves the signal’s statistical properties across all orders. Although this high-dimensional space introduces higher-order nonlinear components, the coefficient B(ω,n) exhibits a decaying trend with increasing order n, ultimately approaching zero. This results in relatively low energy contributions of these higher-order components. Therefore, Equation (7) can be further decomposed into a dominant low-order main term (n=1) and weaker higher-order residual terms (n≥2), expressed as

(12) $$\begin{array}{l} KCWD(t,f)\\ =\int_{-\infty }^{\infty }\int_{-\infty }^{\infty }\kappa_{tf}(t,\tau ,\gamma )A(\omega ,t,\tau )B(\omega ,1)s(t+\tau /2)s(t-\tau /2)e^{-j2\pi f\tau }dud\tau \\ +\int_{-\infty }^{\infty }\int_{-\infty }^{\infty }\kappa_{tf}(t,\tau ,\gamma )A(\omega ,t,\tau )\sum_{n=2}^{\infty }B(\omega ,n){\left(s(t+\tau /2)s(t-\tau /2)\right)}^{2n-1}e^{-j2\pi f\tau }dud\tau \end{array}$$

**Figure 1:**
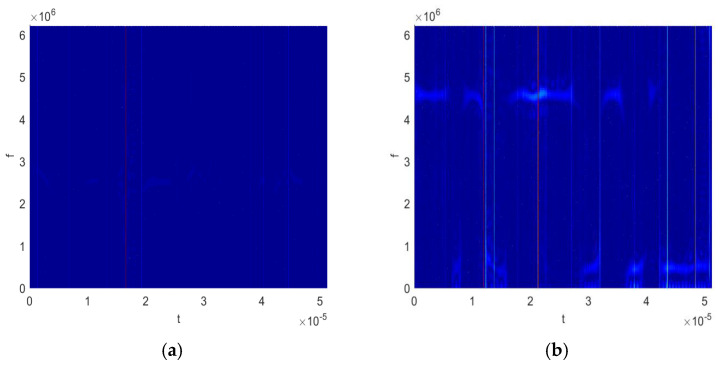

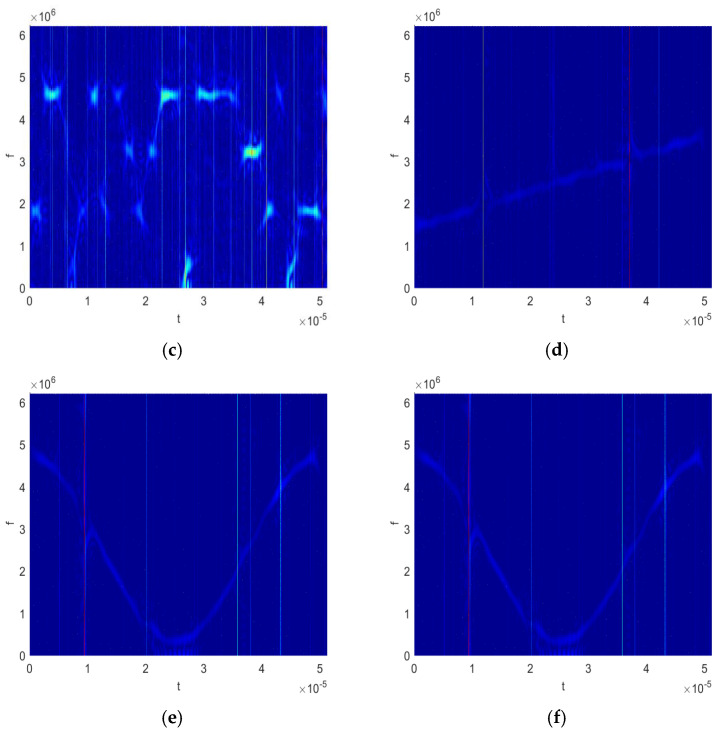
The CWD for the different signals: (**a**) 2PSK; (**b**) 2FSK; (**c**) 4FSK; (**d**) LFM; (**e**) COS-FM; and (**f**) TRI-FM. In the time frequency image, the colors represent signal energy strength. Brighter colors indicate greater energy at that specific time and frequency point.

**Figure 2:**
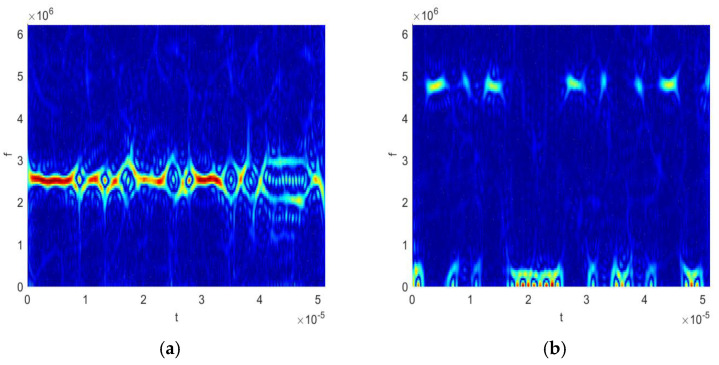

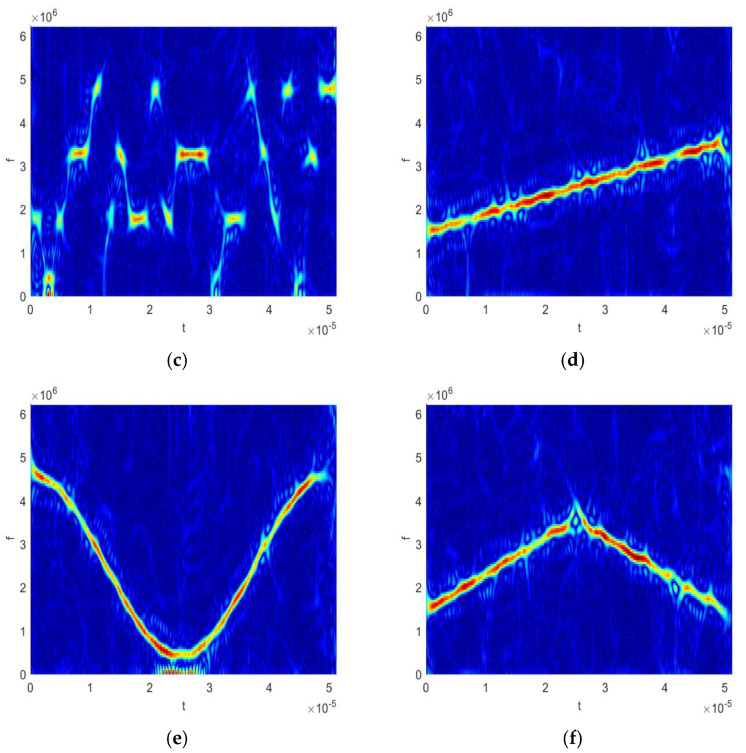
The KCWD for the different signals: (**a**) 2PSK; (**b**) 2FSK; (**c**) 4FSK; (**d**) LFM; (**e**) COS-FM; and (**f**) TRI-FM. In the time frequency image, the colors represent signal energy strength. Brighter colors indicate greater energy at that specific time and frequency point.

The first term in Equation (12) represents the principal component, which can be regarded as a CWD, incorporating weights A(ω,t,τ) and B(ω,n). Consequently, the proposed KCWD effectively characterizes the time–frequency distribution information under impulse noise. It should be noted that in this paper, the hyperbolic tangent kernel function is chosen, which is based on the consideration of the inherent limitations of common kernel functions in suppressing impulse noise. Although the classical Gaussian kernel has a certain noise suppression capability, its second-order correlation component expansion does not correspond to the first harmonic of the signal, resulting in low energy and thus limited feature discriminability. Similarly, polynomial kernels and exponential kernels also have the same limitations. In contrast, the tanh kernel can more effectively focus on the harmonic components carrying key discriminative information, thereby achieving better feature extraction results in this application scenario. Figure 1 and Figure 2 illustrate the CWD and KCWD of six typical signals, including 2PSK, 2FSK, 4FSK, linear frequency modulation (LFM), cosine frequency modulation (COS-FM), and triangular frequency modulation (TRI-FM), under impulse noise. A comparison reveals the following: Figure 1 (CWD) is severely affected by impulse noise, exhibiting distinct striped interference in the time–frequency domain, and Figure 2 (KCWD) demonstrates excellent noise resistance, with the signal’s characteristic distribution remaining clearly discernible. This experimental result closely aligns with the theoretical analysis: the Gaussian decay characteristic of the weight A(ω,t,τ) effectively suppresses impulse noise interference and prevents the generation of stripe noise. Higher-order components maintain low energy levels under the constraint of the coefficient B(ω,n), thus avoiding interference with the signal’s frequency distribution characteristics.

**Figure 3:**
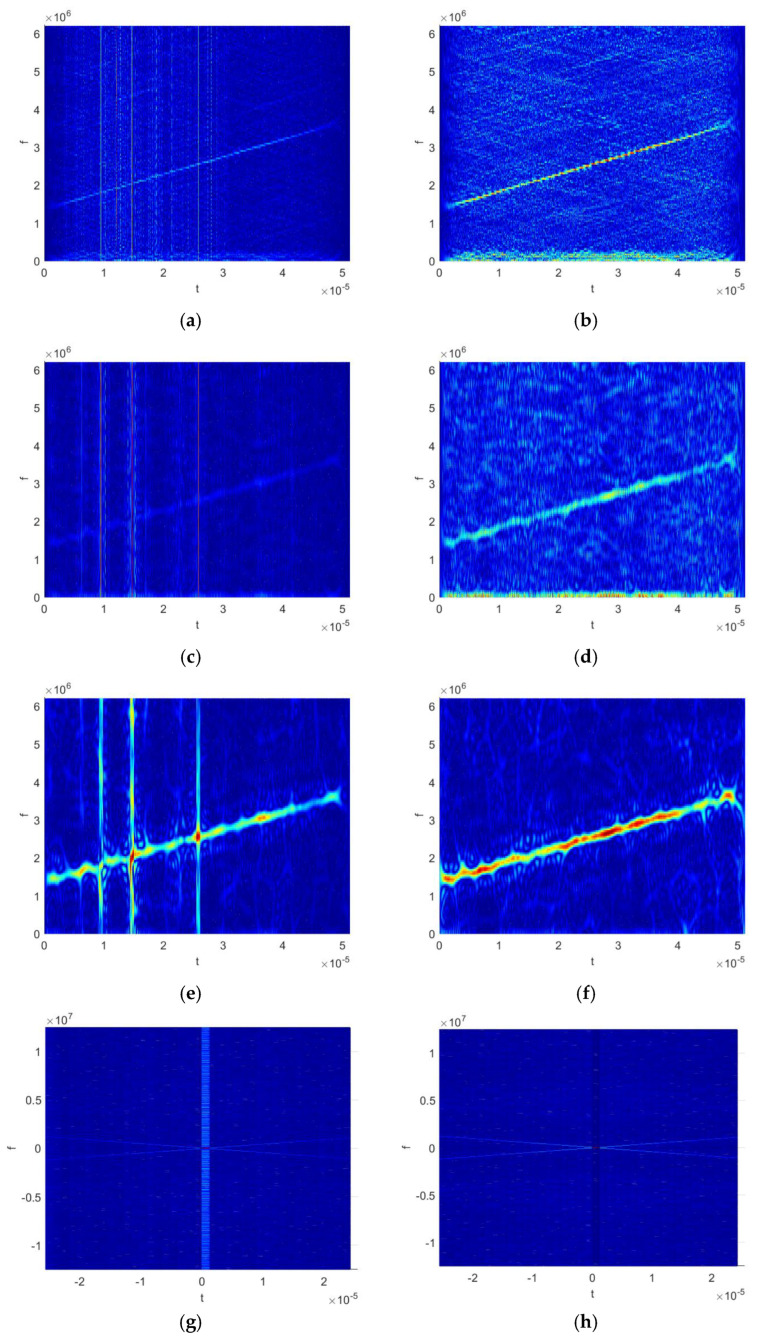
The comparison of eight time–frequency distributions for the LFM signal: (**a**) WVD; (**b**) KWVD; (**c**) PWVD; (**d**) KPWVD; (**e**) SPWVD; (**f**) KSPWVD; (**g**) AF; and (**h**) KAF. In the time frequency image, the colors represent signal energy strength. Brighter colors indicate greater energy at that specific time and frequency point.

In the KCWD method, although current research has only explored the combined application of kernel mapping with the CWD, this approach is obviously applicable to other Cohen-type time–frequency distributions. Building upon this concept, the kernel space mapping technique is further extended to the Wigner–Ville distribution (WVD), pseudo-WVD (PWVD), smoothed pseudo-WVD (SPWVD), and ambiguity function (AF), with corresponding methods named the KWVD, KPWVD, KSPWVD, and KAF, respectively. Their expressions are as follows:

(13) $$KWVD(t,f)=\int_{-\infty }^{\infty }\kappa_{s}\left(s(t+\tau /2),s(t-\tau /2)\right)e^{-j2\pi f\tau }d\tau$$

(14) $$KPWVD(t,f)=\int_{-\infty }^{\infty }h\left(\tau \right)\kappa_{s}\left(s(t+\tau /2),s(t-\tau /2)\right)e^{-j2\pi f\tau }d\tau$$

(15) $$KSPWVD(t,f)=\int_{-\infty }^{\infty }\int_{-\infty }^{\infty }g\left(\mu \right)h\left(\tau \right)\kappa_{s}\left(s(t-\mu +\tau /2),s(t-\mu -\tau /2)\right)e^{-j2\pi f\tau }dud\tau$$

(16) $$KAF(\tau ,f)=\int_{-\infty }^{\infty }\kappa_{s}\left(s(t+\tau /2),s(t-\tau /2)\right)e^{j2\pi f\tau }d\tau$$

where h(τ) and g(μ) represent the time domain window function and frequency-domain window function, respectively. Figure 3 displays the analysis results of a linear frequency modulation (LFM) signal under eight different time–frequency distributions. The four traditional time–frequency analysis methods, including WVD, PWVD, SPWVD, and AF, all exhibit distinct interference fringes, a typical artifact caused by strong pulse noise. In contrast, the four time–frequency distributions enhanced by kernel space mapping, such as KWVD, KPWVD, KSPWVD, and KAF, clearly reveal the signal’s time–frequency ridge structure, with pulse noise components effectively suppressed. This comparative experiment fully demonstrates that kernel space mapping technology possesses universal advantages and can be extended to other Cohen-type time–frequency distributions.

### 3.2. The Multimodal Kernel Harmonic Feature Fusion Network

**Figure 4:**
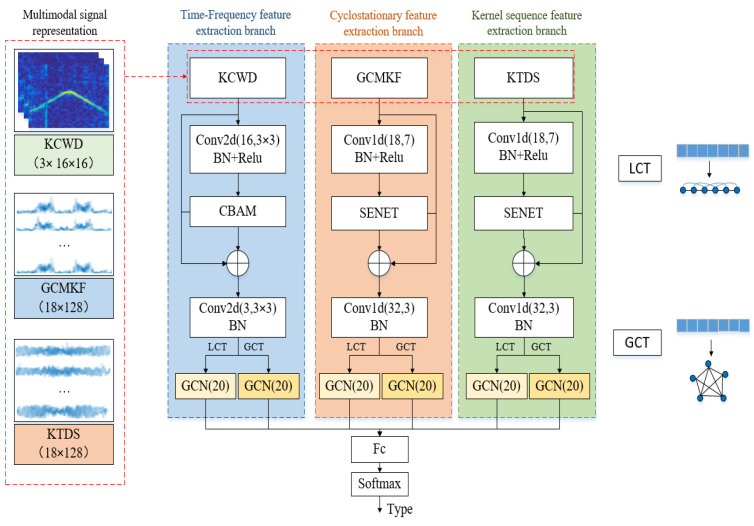
The architecture of the multimodal kernel harmonic feature fusion network.

To address the problem that existing modulation types are becoming increasingly diverse, making it impossible for any single modality to fully characterize signal features, this section constructs the multimodal kernel harmonic feature fusion network (MKHFFN), as shown in Figure 4. This network fuses three types of modal information: time–frequency features (KCWD), cyclostationary features (GCMKF), and kernel space mapping sequences. It employs a graph for the joint local–global modeling of signals, comprehensively enhancing the feature expression ability of the network.

#### 3.2.1. The Time–Frequency Feature Extraction Branch

**Figure 5:**
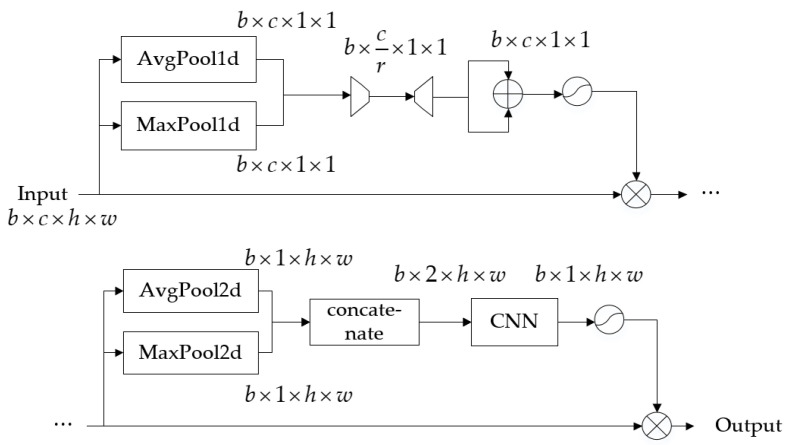
The architecture of the CBAM.

The time–frequency feature extraction branch uses the KCWD as input. Considering that received signals in practical applications typically lack prior information about the impulse noise characteristic index, this method employs three distinct ω values to address the challenge of unknown noise parameters. The attention mechanism is introduced into the network to enable adaptive learning of optimal parameters. Considering that the size of the time–frequency image is too large, it will result in an excessively high number of output feature maps in the first layer of the convolutional layer, thereby significantly increasing the number of parameters in subsequent convolutional layers and graph convolutional layers. If the size is too small, it will reduce the clarity of the time–frequency. Through experiments, the size was downsampled to 16 × 16 using the resize function in MATLAB 9.0.0.341360 (R2016a). Consequently, the signal representation for this branch is 3 × 16 × 16. Subsequently, 2D convolutions are employed for feature extraction, supplemented by batch normalization to accelerate training. Following this, the convolutional block attention module (CBAM) [14] is introduced to guide the network in simultaneously focusing on important channels and key spatial information within the signal, as shown in Figure 5. The CBAM comprises two submodules: channel attention and spatial attention. ln In the channel attention module, spatial dimensions are first compressed through global average pooling and max pooling, yielding two channel values of the same size b×c×1×1. Here, b denotes the batch size and c represents the number of channels. Then, the channel values are fed into a two-layer neural network resembling an autoencoder structure, with the two branches sharing the same weights. The first layer has fewer neurons than c, using the ReLU activation function, while the second layer has c neurons. During this process, channel-to-channel interactions occur. Finally, the two channel attention coefficients are summed and compressed within the range (0, 1) by using the sigmoid function, resulting in the final weight coefficients that represent channel importance, and multiplying these by the original features to obtain new features based on channel attention. In the spatial attention module, features undergo average pooling and max pooling along the channel dimension, yielding two features of the same size b×1×h×w. These spatial features are then convolved to merge channels. After sigmoid compression, the result is multiplied by the original image input to produce new features based on both channel and spatial attention. The attention outputs are connected to the KCWD via residual connections, followed by convolution for channel fusion.

The existing methods are difficult to effectively jointly characterize both the local detail information and global contextual information of signals, limiting the feature expression ability of neural networks. To overcome this bottleneck, graph topology modeling [15] is employed to address the problem. Firstly, convolutional layers are employed to extract deep feature representations with temporal dimensions. Then, these features are structured into graph nodes along the temporal dimension, each endowed with a local connection topology (LCT) and a global connection topology (GCT). Graph convolutional networks (GCNs) are employed to comprehensively extract both the local feature details and global contextual information. The number of graph nodes and their dimensions are 8 and 20, respectively. This approach explicitly constructs topological connections across the temporal dimension, enhancing the modeling of temporal correlations between frequency components. Consequently, the network not only learns static time–frequency distribution patterns but also effectively captures and utilizes dynamic information about how frequencies evolve over time.

#### 3.2.2. The Cyclostationary Feature Extraction Branch

The research [7] proposes the generalized cyclic mean kernel function (GCMKF) based on kernel methods and cyclostationary theory, which suppresses impulse noise and extracts the cyclostationary characteristics of signals. Given the good adaptability of cyclic frequency domain characteristics for digital modulation signal classification, the GCMKF is adopted as the signal representation input for the second branch of the network. In this branch, three near-zero delay values of the GCMKF are selected, and three sets of different ω values are employed to address the issue of unknown noise parameters. Since complex signals are being processed, the GCMKF representation utilizes 18 channels.

**Figure 6:**
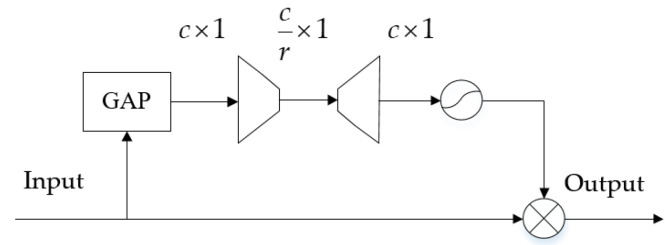
The architecture of the SENET.

Firstly, one-dimensional convolutions are employed for preliminary feature extraction across each channel, followed by batch normalization (BN) to accelerate training convergence. To adaptively select the optimal ω for handling impulse noise, a Squeeze and Excitation Network (SENET) [16] is adopted to focus on important channels, with its structure shown in Figure 6. Global average pooling (GAP) compresses the spatial dimensions of the input, aggregating features from each channel into a scalar value with a global receptive field. Next, a two-layer fully connected network, similar to an autoencoder network structure, compresses and reconstructs channel information to generate weights representing the importance of each feature channel. Finally, these weights are redistributed to the original channels, yielding attention-weighted outputs. To prevent gradient vanishing, the SENET’s attention outputs are connected to the original GCMKF inputs via residual connections, followed by channel fusion through a convolutional layer. Similarly, cyclostationary sequences are modeled with both local and global connection topologies to enhance signal correlation. Finally, a graph convolutional network is employed to extract local and global features.

#### 3.2.3. The Kernel Space Mapping Sequence

It is notable that the kernel function $k_{s}(s(t+\tau /2),s(t-\tau /2))\;$ inherently contains the correlation information of the original data in high-dimensional space. To effectively utilize this information, the kernel time delay slice (KTDS) is introduced as the third branch signal representation for network input. Similar to the cyclostationary feature branch, to address the challenge of unknown noise parameters, three different sets of ω values are employed to generate the KTDS representation. The network model structure for this branch is identical to the previously described cyclostationary feature extraction branch; therefore, its detailed structure is not repeated here.

## 4. Simulation

### 4.1. Parameter Estimation of the LFM Signal Based on the KCWD

**Figure 7:**
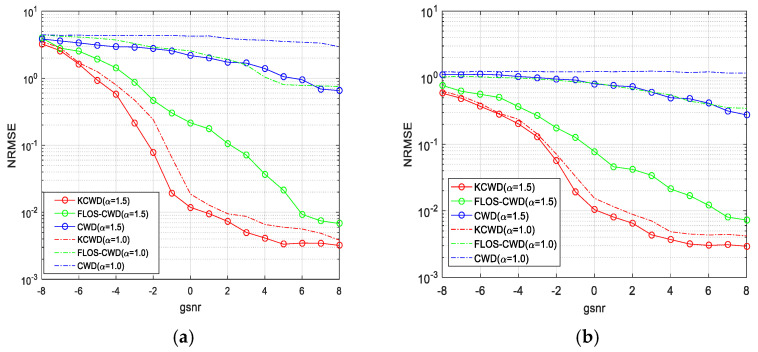
The NRMSE of frequency estimations under the different methods: (**a**) the initial frequency estimation, and (**b**) the frequency modulation slope estimation.

This section analyzes the performance of the KCWD through estimation for the initial frequency and frequency modulation slope of the LFM signal. Figure 7a shows the normalized root mean square error (NRMSE) for estimating the initial frequency of the LFM signals, using methods such as the CWD, FLOS-CWD (CWD based on fractional low-order statistics), and KCWD, based on 300 Monte Carlo experiments. Within the generalized signal-to-noise ratio (GSNR) range of −8 dB to 8 dB, the CWD exhibits a significantly higher NRMSE than the other two methods. This is because the CWD fundamentally relies on the second-order statistics of the signal. In impulse noise environments, where noise moments are infinite, the LFM time–frequency ridge information is destroyed, leading to a sharp deterioration in parameter estimation performance. In contrast, the KCWD exhibits the smallest NRMSE and demonstrates optimal robustness against impulse noise, because the kernel function projects the original signal into a high-dimensional feature space via a nonlinear mapping. Within this space, the impact of impulse noise is effectively suppressed, allowing the signal’s time–frequency characteristics to be preserved, thereby enhancing parameter estimation accuracy. The performance of the FLOS-CWD is between the KCWD and CWD. It uses fractional-order statistics (FLOSs) instead of second-order statistics, which can suppress large-amplitude impulse noise to some extent. However, its effectiveness depends on the precise selection of the fractional order, and its ability to suppress dense or small-amplitude impulse noise is limited. So, its performance is inferior to the KCWD.

Additionally, as the parameter α, characterizing the probability of impulse noise occurrence, decreases, the NRMSE of all three methods increases, leading to a deterioration in parameter estimation performance. This is because, as the parameter α decreases, pulses occur more frequently, enhancing the non-Gaussianity of the signal. However, the KCWD exhibits relatively minor NRMSE variation with changes in α, indicating that its kernel function approach effectively handles varying degrees of impulse noise. This robustness demonstrates significant advantages in dynamic noise environments.

Figure 7b shows the NRMSE of the three methods for estimating the frequency modulation slope of the LFM signal. The simulation results exhibit a consistent trend with the initial frequency estimation: the KCWD delivers the optimal performance and strongest robustness in estimating the modulation slope. This further validates the effectiveness of the KCWD method in suppressing impulse noise through kernel functions in high-dimensional space.

### 4.2. Classification Accuracy Under Different Models and Different Inputs

#### 4.2.1. Dataset

The dataset was generated through MATLAB simulation and includes six types of signals: BPSK, 2FSK, 4FSK, LFM, COS-FM, and TRI-FM. The key parameters are as follows: a sampling frequency of 12.5 MHz, a sampling duration of 40.96 μs, signal frequency ranging from 300 Hz to 300 kHz, and a randomly set bandwidth. The generalized signal-to-noise ratio (GSNR) ranges from −12 dB to 12 dB, with a step size of 2 dB. The characteristic exponent of the noise is 1.5. The sample size for each signal at each GSNR level is 1000, and it is divided into a training set and a test set in an 8:2 ratio.

#### 4.2.2. Comparison of Recognition Accuracy

This experiment evaluates the performance of the proposed method by comparing the recognition rates under different network architectures and signal representations. The specific experimental setup is as follows: CNN-A, CLDNN-A, and FEA-Transformer-A are used as the baseline models, with their input features being the same as those in the MKHFFN, all being multi-modal harmonic features, while MKHFFN-B, CNN-B, CLDNN-B, and FEA-Transformer-B use the original multi-modal features (including time–frequency features, cyclostationary features, and related features) that have not undergone kernel space mapping as the input. The baseline models are derived from references [17,18,19]. Additionally, to deeply analyze the contributions of each module in the MKHFFN, a series of ablation experiments is constructed to compare the models. MKHFFN-C, MKHFFN-D, and MKHFFN-E represent the removal of time–frequency features, cyclostationary features, and kernel sequence features from the MKHFFN. MKHFFN-F and MKHFFN-G, respectively, removing the local graph convolution transformation (LCT) and global graph convolution transformation (GCT) modules. MKHFFN-H removes both the LCT and GCT modules from the MKHFFN.

**Figure 8:**
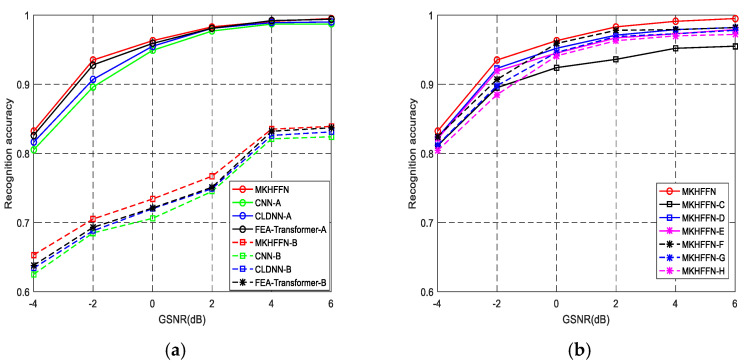
Recognition accuracy comparison between MKHFFN and (**a**) existing AMC methods, and (**b**) its varieties.

As shown in Figure 8a, the proposed MKHFFN method consistently outperforms models such as CNN-A, CLDNN-A, and FEA-Transformer-A in terms of recognition performance. This advantage mainly stems from the graph structure introduced by the MKHFFN, which can jointly model both the local and global aspects of the signal, thereby achieving stronger feature representation capabilities. CLDNN-A outperforms CNN-A due to the effective time information capture ability of its LSTM module; however, its global modeling ability still lags behind that of the graph convolution mechanism adopted by the MKHFFN. FEA-Transformer-A improves the recognition rate through its multi-layer self-attention mechanism, but still fails to surpass the MKHFFN. Additionally, the recognition rates of MKHFFN-B, CNN-B, CLDNN-B, and FEA-Transformer-B are lower than those of their corresponding A-type models, which validates that the signal representation based on kernel space mapping can effectively suppress impulse noise.

From the ablation experiment results in Figure 8b, it can be seen that the performance of MKHFFN-C, MKHFFN-D, and MKHFFN-E is all lower than that of the complete version of the MKHFFN, proving that the three modal features are complementary. Among them, the performance of MKHFFN-C has decreased the most significantly, highlighting the crucial role of the corresponding modal for characterizing the widely existing time-varying signals in the data set. At the same time, the performance of MKHFFN-F, MKHFFN-G, and MKHFFN-H is also generally lower than that of the complete model, indicating that the graph modeling module as a whole enhances the network’s feature extraction ability. Particularly, the recognition rate of MKHFFN-G is lower than that of MKHFFN-F, suggesting that the GCT global graph modeling branch, compared to the local graph structure, is more crucial for improving the feature expression ability.

**Figure 9:**
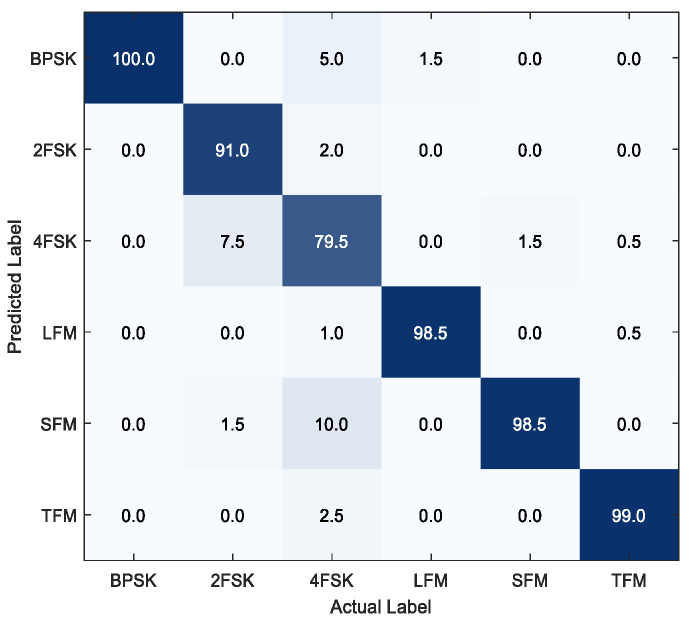
Confusion matrix.

Figure 9 presents the confusion matrix results under a −2 dB generalized signal-to-noise ratio. As a phase-modulated signal, the BPSK exhibits significant differences from the five frequency-modulated signals in the feature space, achieving a recognition rate of 100%. All three continuous-wave modulation signals of the LFM, COS-FM, and TRI-FM achieved recognition rates over 98.5%, with a low degree of confusion. This advantage primarily stems from two aspects: Firstly, the MKHFFN employs a multimodal signal representation method that effectively integrates multi-source features, such as time–frequency, cyclostationary, and kernel mapping sequences, significantly enhancing feature diversity and information richness. Secondly, the proposed method utilizes local and global connectivity graph modeling to fully characterize the signal features, particularly the differences in frequency variations across the five frequency-modulated signals. The 4FSK signals exhibit varying degrees of confusion with other signals. This occurs because, under low signal-to-noise ratio conditions, the multiple frequency transition points in the 4FSK modulation are susceptible to noise contamination, leading to frequency blurring and energy diffusion in the time–frequency image.

#### 4.2.3. Comparison of Computational Complexity

Table 1 comprehensively compares the computational complexity of the proposed MKHFFN model, its variants, and several benchmark models. All simulation experiments were conducted on a unified hardware platform, comprising an Intel(R) Core(TM) i7-7700 CPU (Intel Corporation, Santa Clara, CA, USA) and a NVIDIA GeForce GTX 1070 GPU (NVIDIA Corporation, Santa Clara, CA, USA), to ensure the comparability of the results. The MKHFFN is roughly equivalent to CLDNN-A in computational complexity. The MKHFFN outperforms CNN-A in terms of parameters and training time, mainly due to the node information aggregation and multiple matrix operations involved in its graph structure. However, compared to FEA-Transformer-A, the training time of the MKHFFN is only 46% of that of FEA-Transformer-A, because the high computational burden brought by the multi-layer self-attention mechanism in the Transformer is involved. In the ablation models, MKHFFN-H, after completely removing the graph modeling module, has a parameter reduction to a level similar to CNN-A, which reflects that the graph modeling mechanism indeed introduces additional computational overhead. Compared to MKHFFN-F (which removes the LCT module), MKHFFN-G (which removes the GCT module) has a slightly faster training speed, indicating that the computational complexity of the GCT module is slightly higher than that of the LCT module.

In conclusion, the complete MKHFFN model has improved recognition performance within an acceptable computational cost range.

## 5. Conclusions

This paper proposes an automatic recognition method based on the multimodal kernel harmonic feature fusion network (MKHFFN) to solve the degradation of modulation recognition performance in pulsed noise environments. First, the KCWD is proposed to effectively suppress impulse noise and enhance the distinguishability of time–frequency features. On this basis, the MKHFFN is constructed to comprehensively extract time–frequency features, cyclostationary features, and kernel space mapping sequence features, overcoming the limitations of single-modality information in fully characterizing signal modulation characteristics. Finally, a graph-based structure enables joint local–global modeling of the signal, enhancing the feature expression capability of the model for modulation patterns. The simulation results show that this method achieves a recognition rate of 93.5%, at a low signal-to-noise ratio of −2 dB. The KCWD can be applied not only to modulation recognition tasks but also to various signal processing tasks in an impulse noise environment, such as signal detection and parameter estimation. In addition, the MKHFFN incorporates the attention mechanism to adaptively select optimal parameters, effectively avoiding dependence on noise characteristic exponent estimation. To enhance the generalization and robustness of the model, future work will focus on actual channel conditions, such as Gaussian and impulse-mixed noise, as well as multipath fading. Concurrently, the network model’s transfer learning mechanisms under varying noise and channel conditions will be investigated to improve its adaptability in practical systems.

## Figures and Tables

**Table 1:** Comparison of computational complexity.

	Parameters (k)	Training Time (s/epoch)
MKHFFN	206	35
CNN-A	167	15
CLDNN-A	211	32
FEA-Transformer-A	323	76
MKHFFN-F	176	28
MKHFFN-G	159	24
MKHFFN-H	135	17

## Data Availability

The data that support the findings in this study are available from the corresponding author upon reasonable request.
